# AZGP1 deficiency promotes angiogenesis in prostate cancer

**DOI:** 10.1186/s12967-024-05183-x

**Published:** 2024-04-24

**Authors:** Ru M. Wen, Zhengyuan Qiu, G. Edward W. Marti, Eric E. Peterson, Fernando Jose Garcia Marques, Abel Bermudez, Yi Wei, Rosalie Nolley, Nathan Lam, Alex LaPat Polasko, Chun-Lung Chiu, Dalin Zhang, Sanghee Cho, Grigorios Marios Karageorgos, Elizabeth McDonough, Chrystal Chadwick, Fiona Ginty, Kyeong Joo Jung, Raghu Machiraju, Parag Mallick, Laura Crowley, Jonathan R. Pollack, Hongjuan Zhao, Sharon J. Pitteri, James D. Brooks

**Affiliations:** 1 Department of Urology, Stanford University School of Medicine, Stanford, CA 94305 USA; 2 Department of Molecular and Cellular Physiology, Stanford University School of Medicine, Stanford, CA 94305 USA; 3 Department of Radiology, Stanford University School of Medicine, Stanford, CA 94305 USA; 4 Canary Center at Stanford for Cancer Early Detection, Stanford University School of Medicine, Stanford, CA 94305 USA; 5 GE HealthCare Technology and Innovation Center, Niskayuna, NY 12309 USA; 6 Department of Computer Science and Engineering, The Ohio State University, Columbus, OH 43210 USA; 7 Department of Biochemistry, Stanford University School of Medicine, Stanford, CA 94305 USA; 8 Howard Hughes Medical Institute, Stanford University School of Medicine, Stanford, CA USA; 9 Department of Pathology, Stanford University School of Medicine, Stanford, CA USA

**Keywords:** AZGP1, Angiogenesis, Tumor microenvironment, Fibrosis, Prostate cancer

## Abstract

**Background:**

Loss of AZGP1 expression is a biomarker associated with progression to castration resistance, development of metastasis, and poor disease-specific survival in prostate cancer. However, high expression of AZGP1 cells in prostate cancer has been reported to increase proliferation and invasion. The exact role of AZGP1 in prostate cancer progression remains elusive.

**Method:**

AZGP1 knockout and overexpressing prostate cancer cells were generated using a lentiviral system. The effects of AZGP1 under- or over-expression in prostate cancer cells were evaluated by in vitro cell proliferation, migration, and invasion assays. Heterozygous AZGP1^±^ mice were obtained from European Mouse Mutant Archive (EMMA), and prostate tissues from homozygous knockout male mice were collected at 2, 6 and 10 months for histological analysis. In vivo xenografts generated from AZGP1 under- or over-expressing prostate cancer cells were used to determine the role of AZGP1 in prostate cancer tumor growth, and subsequent proteomics analysis was conducted to elucidate the mechanisms of AZGP1 action in prostate cancer progression. AZGP1 expression and microvessel density were measured in human prostate cancer samples on a tissue microarray of 215 independent patient samples.

**Result:**

Neither the knockout nor overexpression of AZGP1 exhibited significant effects on prostate cancer cell proliferation, clonal growth, migration, or invasion in vitro. The prostates of AZGP1^−/−^ mice initially appeared to have grossly normal morphology; however, we observed fibrosis in the periglandular stroma and higher blood vessel density in the mouse prostate by 6 months. In PC3 and DU145 mouse xenografts, over-expression of AZGP1 did not affect tumor growth. Instead, these tumors displayed decreased microvessel density compared to xenografts derived from PC3 and DU145 control cells, suggesting that AZGP1 functions to inhibit angiogenesis in prostate cancer. Proteomics profiling further indicated that, compared to control xenografts, AZGP1 overexpressing PC3 xenografts are enriched with angiogenesis pathway proteins, including YWHAZ, EPHA2, SERPINE1, and PDCD6, MMP9, GPX1, HSPB1, COL18A1, RNH1, and ANXA1. In vitro functional studies show that AZGP1 inhibits human umbilical vein endothelial cell proliferation, migration, tubular formation and branching. Additionally, tumor microarray analysis shows that AZGP1 expression is negatively correlated with blood vessel density in human prostate cancer tissues.

**Conclusion:**

AZGP1 is a negative regulator of angiogenesis, such that loss of AZGP1 promotes angiogenesis in prostate cancer. AZGP1 likely exerts heterotypical effects on cells in the tumor microenvironment, such as stromal and endothelial cells. This study sheds light on the anti-angiogenic characteristics of AZGP1 in the prostate and provides a rationale to target AZGP1 to inhibit prostate cancer progression.

**Supplementary Information:**

The online version contains supplementary material available at 10.1186/s12967-024-05183-x.

## Background

The large discrepancy in prostate cancer (PCa) incident rates, estimated at 288,300 in 2023 in the U.S., and death rates, estimated to be 34700, demonstrates the need for prognostic biomarkers to select patients for observation or aggressive treatment [1]. In 2004, our group demonstrated that loss of expression of zinc-alpha-2-glycoprotein (AZGP1) was a candidate biomarker of PCa aggressiveness [2]. Since that time, several large-scale studies have validated that loss of expression of AZGP1 in PCa is a strong predictor of poor outcomes (including recurrence, metastasis, progression to castrate resistance, and PCaspecific death), as well as providing predictive value independent of clinical and pathological parameters (such as tumor grade, stage, and pre-operative serum prostate specific antigen (PSA) levels) [3–12]. AZGP1 is part of a commercial assay that has been used clinically to predict PCa outcomes in several clinical settings, most commonly in selection of patients for active surveillance [13]. In addition, AZGP1 loss has been found to be a predictor of poor outcomes in several malignancies including gastric cancer [14], esophageal squamous cell carcinoma [15], liver cancer [16], and bladder cancer [17].

While many prognostic biomarkers play roles in cancer progression, such as by driving proliferation or metastasis, the role of AZGP1 in cancer aggressiveness remains unclear for PCa. In liver cancer, AZGP1 has been found to inhibit the TGF-β1-ERK2 pathway, PTEN/Akt pathway, the CD44 signaling pathway [18, 19], and epithelial-to-mesenchymal transition (EMT). thereby blocking lung metastasis [18]. However, in gastric cancer, overexpression of AZGP1 has been reported to accelerate apoptosis and inhibit growth through modulation of the mTOR/PTEN signaling pathway [20]. In PCa, a single study has reported that AZGP1 can affect proliferation and invasion of PCa cells [21]. Strikingly, over-expression of AZGP1 in PCa cells increased aggressive behavior, contradicting the many clinical studies demonstrating that loss of protein and RNA expression of AZGP1 are associated with cancer progression and mortality [3, 8, 10]. Given the limited investigations into the mechanisms through which AZGP1 affects cancer behavior, its role remains poorly defined. To better understand whether AZGP1 plays a direct role in PCa progression, we investigated the effects of modulating expression of AZGP1 in vitro and in vivo.

## Materials and methods

### Cell culture

The human PCa cell lines PC3, DU145, LNCaP, C42B,, 22Rv1 and human umbilical vein endothelial cells (HUEVEC) were obtained from American Type Culture Collection (Manassas, VA). PC3, LNCaP, C42B, and 22Rv1 cells were grown in RPMI-1640 medium (Invitrogen Inc., CA, USA) supplemented with 10% fetal bovine serum (FBS) and 100 U/ml penicillin/streptomycin. DU145 were cultured in Dulbecco’s modified Eagle medium (Invitrogen Inc.) containing 10% FBS and 100 U/ml penicillin/streptomycin. HUVEC cells were grown in human large vessel endothelial cell basal medium (catalog no. M200500, Thermofisher Scientific) with low serum growth supplement (catalog no. S00310, ThermoFisher Scientific). The human LuCaP147 patient derived xenograft (PDX) was generously provided by Dr. Eva Corey [22]. Human LuCaP147 spheroids were maintained in StemPro^®^ hESC SFM (Invitrogen Inc.) medium supplemented with 10 nM of R1881 and 2 μM of Y-27632 as previously described [23]. All cells were maintained in a humidified 5% CO_2_ incubator at 37 °C and periodically monitored for mycoplasma contamination by polymerase chain reaction (PCR) PCR. LuCaP147 spheroids passaged less than 15 times and PCa cell lines with passages fewer than 20 were used in this study.

### Plasmids, antibodies, chemicals

The control sgRNA (catalog no. K010) and sgAZGP1 plasmids (sgAZGP1-T1: AGCCAAGGGCCTGAAACGCG and sgAZGP1-T2: GACAGGAAGTCTCAGCCCAT, catalog no. 12907111) were obtained from Applied Biological Materials USA Inc. (Ferndale, WA, USA). The control GFP (catalog no. LVCV-0) and AZPG1 cDNA (catalog no. HG13242-ACGLN) were procured from Sino Biologicals US Inc. (Houston, TX, USA). An anti-AZGP1 antibody was purchased from OriGene (Rockville, MD, USA). Anti-Ki67 (catalog no. 12202) and secondary antibodies were purchased from Cell Signaling (Danvers, MA, USA). Anti-CD31 (catalog no. ab182981) was purchased from Abcam (Cambridge, UK). Polybrene and puromycin were purchased from Sigma-Aldrich (St. Louis, MO, USA).

### Lentiviral production

The sgAZGP1 and AZGP1 cDNA plasmids were amplified using a E.Z.N.A.^®^ Plasmid DNA Maxi Kit (Omega Bio-tek, Norcross, GA, USA) following manufacturer’s instructions, and used to transfect 293 T cells using third generation lentiviral system with MDL, VSV, and Rev as described previously[24]. The medium was changed after 12 h and cells were maintained for additional 48 h. The supernatant was collected by centrifugation, filtered using a 0.45 µm membrane, and precipitated with PEG-it^™^ Virus Precipitation Solution (System Biosciences, Palo Alto, CA, USA). The lentiviruses were collected and used to transfect the PCa cells.

### Generation of AZGP1 knockout and overexpressing cells

AZGP1 knockout cells were generated by sgAZGP1 lentiviral transduction. Specifically, LNCaP cells were transduced with sgAZGP1 lentiviral particles for 72 h and cells were washed with PBS (3X) followed by puromycin selection for 5 days. The LuCaP147 cells were transduced in a similar manner except that no puromycin selection was performed due to its extreme toxicity to the spheroids. Similarly, PC3, DU145, LNCaP, and LuCaP147 cells were transduced with AZGP1 cDNA lentiviral particles to obtain AZGP1 overexpressing cells.

### Reverse-transcription polymerase chain reaction (RT-PCR) and real-time quantitative RT-PCR (RT-qPCR)

The total RNA was isolated using a RNeasy Mini kit (Qiagen, Redwood City, CA, USA) according to manufacturer’s instructions. The complementary DNA (cDNA) was obtained using the High-Capacity cDNA Reverse Transcription Kit (ThermoFisher Scientific, Waltham, MA, USA). Quantitative RT-PCR was performed using a standard protocol from the SYBR Green PCR Kit (ThermoFisher Scientific) using the StepOnePlus Real-Time PCR System (Applied Biosystems, Waltham, MA). The primers used were: AZGP1, forward 5′- AACGACAGTAACGGGTCTCAC-3′; AZGP1, reverse 5′-TTGGTTATCTGGGCTGCTGG-3′; β-actin, forward 5′-GATCATTGCTCCTCCTGAGC-3′; β-actin, reverse 5′-CGTCATACTCCTGCTTGCTG-3′.

### Proliferation assays

LuCaP147 control vector and LuCaP147 AZGP1-OV, LuCaP147 control sgRNA, LuCaP147 sg-AZGP1-T1 and LuCaP147 sg-AZGP1-T2 spheroids were dissociated into small clusters using Accutase (Cat# AT104, Innovative Cell Technologies, San Diego, CA, USA). Cells (2 × 10^4^ cells/well) were transferred into 96-well plates and cultured in the medium for 9 days. Cell growth was measured with CellTiter-Glo^®^ 3D Cell Viability Assay (catalog no. G96821, Madison, WI, USA) according to the manufacturer's instructions as a readout for LuCaP147 spheroid proliferation.

LNCaP control vector and LNCaP AZGP1-OV, 22Rv1 control vector and 22Rv1 AZGP1-OV, LNCaP control sgRNA, LNCaP sg-AZGP1-T1 and LNCaP sg-AZGP1-T2 (1500 cells/well) were plated in 12-well plates and cultured in triplicate as described above. Cells were trypsinized and counted manually on days 2, 4, and 7.

HUVEC cells were cultured (800 cells/well) in 96-well plates and treated with or without AZGP1 protein (1, 10 µg/mL, Catalog no. 13242-H08H, Sino Biological) in the medium for 7 days. Cell growth was measured with CyQUANT^™^ LDH Cytotoxicity Assay (catalog no. C20300, Thermofisher Scientific) according to the manufacturer's instructions.

### Colony formation assay

LNCaP control vector and LNCaP AZGP1-OV, LNCaP control sgRNA, LNCaP sg-AZGP1-T1 and LNCaP sg-AZGP1-T2, 22Rv1 control and 22Rv1 AZGP1-OV (4000 cells/well) were plated into 6-well plates and cultured for 11 days, with media exchanges every three days. Colonies were washed with PBS (2X) and fixed with 4% (w/v) paraformaldehyde for 10 min at room temperature. The fixed colonies were stained with 0.05% (w/v) crystal violet for 10 min and then washed with PBS (5X). The relative colony numbers were counted using Image J.

### Cell migration detection by wound-healing assay

PC3 control vector, PC3 AZGP1-OV, DU145 control vector and DU145 AZGP1-OV (2 × 10^6^ cells/well) were plated in 6-well plates. The next day, when cells at the bottom of the plate were close to 100% confluency, the plates were scratched with a 200 µL pipette tip. Pictures of the scratched areas were taken on day 0 and day 1 to measure the width of the wounded gaps. The degree of wound closure for each replicate, expressed as a percentage, was determined using Image J.

### Transwell migration assay

PC3 control vector and AZGP1-OV cells (1 × 10^5^ cells), HUEVEC (1 × 10^**5**^ cells) were seeded in serum-free media onto 24-well transwell inserts (Transwell™ Permeable Polyester Membrane Inserts with 8 µm-pore size membrane) (Corning, Corning, NY, USA). HUVEC cells were treated with or without AZGP1 protein (10 µg/ml). All the cells were incubated for 24 h. The remaining cells on top of the membrane were removed from the transwell insert using cotton-tipped applicator. The transwell inserts together with cells that had migrated through the membrane to the bottom of the inserts, and cells that had migrated to the bottom of the wells were then fixed with 4% PFA and stained with 0.01% crystal violet, followed by thorough washing with PBS and air drying for 10–15 min. The cells that had migrated through the membrane and into the bottom of the wells were imaged and counted by Image J.

### Transwell invasion assay

BD Matrigel™ (30 µl, San Jose, CA, USA) was added to a 24-well transwell insert (8 µm-pore size membrane) and solidified in a 37 °C incubator for 30 min to form a thin layer on top of the transwell membrane. PC3 control vector and AZGP1-OV (2 × 10^5^ cells) were seeded in serum-free media on top of the transwell insert and incubated for 24 h. Cells that had migrated into the Matrigel layer were fixed in situ and stained with 0.01% crystal violet solution. The stained cells were imaged and counted manually.

### In vitro* angiogenesis assay*

Matrigel (50 µL, Corning) was added to 96-well plate plates and incubated at 37 ºC for 1 h to form a gel layer. HUVEC cells in media (1.5 × 10^4^ cells) were added to the surface of the Matrigel layer. The cells were treated with AZGP1 protein (10 ug/ml) or media control and incubated at 37 ºC for 24 h. The cells were stained using calcein AM (C3099, ThermFisher Scientfic) and imaged microscopically.

### PCa xenograft studies

NSG (NOD/LtSz-SCID IL-2Rγnull) mice were obtained from the Jackson Laboratory and were housed at the Veterinary Service Center of Stanford University. The use of animals in the study adhered to the guidelines and regulations established by the Institutional Animal Care and Use Committee (IACUC) of Stanford University. PC3 control vector, PC3 AZPG1-OV, DU145 control and DU145 AZGP1-OV (1 × 10^6^ cells), 22Rv1 control and 22Rv1 AZGP1-OV (2 × 10^6^ cells) were suspended in 50% Matrigel (100 µL) and were injected subcutaneously into the dorsal flank of 10–12 week-old NOD.Cg-Prkdc scid Il2rg tm1Wjl /SzJ (NSG) mice (The Jackson Laboratory, Sacramento, CA, USA). The tumor size was measured every 3–4 days using a Vernier caliper, and tumor volume was calculated as V = 1/2 × L × W^2^. At the end of experiment, mice were sacrificed, and the tumors were collected. Half of the tissues were formalin-fixed and paraffin-embedded and the other half snap frozen for proteomic analysis by mass spectrometry.

### Western blots

PC3 control vector and AZGP1-OV cells, DU145 control vector and DU145 AZGP1-OV cells, LNCaP control vector and LNCaP AZGP1-OV, LNCaP control sgRNA, LNCaP sg-AZGP1-T1 and LNCaP sg-AZGP1-T2, LuCaP147 control vector and LuCaP147 AZGP1-OV, LuCaP147 control sgRNA, LuCaP147 sg-AZGP1-T1 and LuCaP147 sg-AZGP1-T2 cells were lysed in RIPA lysis buffer containing 1X protease and phosphatase inhibitors (Thermo Fisher Scientific). PC3 control tumors and AZGP1-OV tumors were minced to small pieces and then dissolved in RIPA buffer containing 1X protease and phosphatase inhibitors with probe sonicator (Fisher Scientific). Protein concentration was measured by BCA assay (Thermo Fisher Scientific). Protein samples in 1X Laemmli sample buffer (Bio-rad, Hercules, CA, USA) were heat-denatured at 95 °C for 10 min. The protein lysates were loaded and separated by SDS-PAGE, transferred onto a nitrocellulose membrane, and blotted with primary antibodies including anti-β actin (a00702, 1:1000 dilution, Genescript, Piscataway, New Jersey, USA), anti-AZGP1 (TA8121185, 1:1000 dilution, OriGene Technologies, Inc., MD, USA), and anti-AR (ab133273, 1:1000 dilution, Abcam, Boston, MA, USA) and secondary anti-mouse IgG conjugated to HRP (7076, 1:5000 dilution, Cell Signaling Technology, Inc, Danvers, MA, USA) or anti-rabbit IgG HRP conjugated antibodies (7074, 1:5000 dilution, Cell Signaling Technology, Inc, Danvers, MA, USA). Western blot development and detection were performed using ECL^™^ Western Blotting Substrate (GERPN2106, Cytiva, Marlborough, MA, USA).

### AZGP1 knockout mice breeding

A heterozygous AZGP1 knockout male mouse (EM:02573, B6.129P2-Azgp1^tm1.1Sbah^/Orl)[25] was obtained from European Mouse Mutant Archive (EMMA). Heterozygous AZGP1 knockout female mice were obtained by breeding the heterozygous AZGP1 knockout male mouse with wild type female C57BL/6 mice. Homozygous AZGP1 knockout mice were obtained by cross-breeding male and female heterozygous AZGP1 knockout mice, and the offspring were genotyped as described above. All mice used here were of C57BL/6 background. The genotypes of the offspring were determined by PCR using the Kapa Mouse Genotyping kit (Cliniscience, Nanterre, France) with AZGP1 primers (AZGP1-forward 5′- ACTCTGTGCCAGGCTCAGGTG-3′, AZGP1-reverse 5′-ACCACAGGTCAGTCTGATTAC-3′).

### Hematoxylin and Eosin staining

The tissue sections underwent a sequential immersion process in a series of solvents: Clearify (SKU #: CACLELT, Statlab LLC, Texas TX) for 5 min (2X), 100% ethanol, 95% ethanol and 75% ethanol for 2 min each (2X). The sections were stained with Harris hematoxylin for 5 min, followed by 5–6 quick dips in acid alcohol (0.3%) and Scott's Tap Solution for 3 min, Eosin solution for 30 s. Dehydration was performed by immersing the sections in 95% alcohol, 100% alcohol, and xylene for 2 min each (2X). Finally, the sections were air dried and mounted with a coverslip.

### Immunohistochemistry staining

The tissue sections were baked at 60 °C for 60 min and sequentially immersed in clearify, 100% ethanol, 95% ethanol, and 70% ethanol (5 min). For antigen retrieval, the tissue sections were boiled in 0.01 M citrate buffer (pH 6.0) for 20 min, and slides were blocked in 5% goat serum and 5% BSA in PBS for 1 h. After that, the sections were incubated with a primary antibody AZGP1 (TA8121185, 1:100 dilution, OriGene Technologies, Inc., MD, USA), CD31(1:100, ab182981, Abcam, Boston, MA, USA) in PBS containing 0.5% BSA overnight at 4 °C. The following day, the sections were washed with TBST(3X) and treated with 0.3% H_2_O_2_ in distilled water to block endogenous peroxidase activity. Subsequently, a secondary antibody was applied in PBS containing 0.05% BSA. DAB solution (catalog No. SK-4100, Vector Laboratories) was used to develop the tissue slides. The slides were stained with Hematoxylin for 5 min, followed by immersion in NaHCO_3_ solution for 3 min. The slides were then immersed in 75%, 95%, and 100% ethanol, and xylene, for 5 min each (2X).

### Immunofluorescence staining

All the steps were performed in the same manner as immunohistochemical staining up to primary antibody (anti-CD31, 1:200, catalog no. 77699S, Cell Signaling Inc) incubation at the end of day 1. The following day, the slides were washed with TBST (3X) and then incubated with secondary antibodies (catalog no. ab150077, Abcam) diluted with 1 × PBS containing 0.05% BSA (1:400) for 1 h at room temperature. The slides were washed with TBST (3X) and mounted with mounting medium with DAPI (catalog no. ab104139) and sealed with clear nail polish for image.

### Sirius red staining

The tissue sections were baked at 60 °C for 60 min and hydrated by sequential immersion in clearify, 100% ethanol, 95% ethanol, and 70% ethanol for 2 times (5 min each). Slides were stained with Hematoxylin for 1 min, and immersed in NaHCO_3_ solution (50 mM) for 3 min. After washing for 10 min in running tap water, the slides were stained with picro-sirius red (catalog no. ab246832, Abcam) for one hour, and washed twice with acidified water (0.5% acetic acid). The slides were dehydrated in 100% ethanol (5 min, 3X) and xylene (5 min, 2X) and were mounted and imaged.

### Proteomic analysis

Proteins were extracted from control-GFP and AZPG1-OV PC3 tumors by adding 800 µl of lysis buffer containing 1.5% SDS buffer with protease inhibitor and homogenized using a probe sonicator (Fisher Scientific). The supernatant was collected after centrifuged at 14,000 g for 10 min at 4 °C and proteins were evaluated by BCA protein assay according to manufacturer’s instructions (Thermo Fisher Scientific). The protein was reduced using Tris(2carboxyethyl) phosphine (10 mM, TCEP, Sigma-Aldrich), alkylated iodoacetamide (15 mM, Acros Organics), and digested with trypsin (Thermo Fisher Scientific). The resulting tryptic peptides were reconstituted in 0.1% formic acid (Fisher Scientific), and loaded into a C18 trap column (Thermo Fisher Scientific) for LC/MS analysis on an Orbitrap Trybrid Eclipse mass spectrometer (Thermo Fisher Scientific). The flow rate was 0.6 µl/min. Eluted peptides were ionized with a Nanospray Flex Ion Source (1.8 kV, Thermo Fisher Scientific) coupled to an LTQ Orbitrap Elite mass spectrometer (Thermo Fisher Scientific). The flow rate is 5 µL/min. 0.1% formic acid in water was used as mobile phase A and 0.1% formic acid in acetonitrile was used as mobile phase B. The column was heated to 65 °C by a column heater (PST Phoenix S&T). The gradient program was set to 2% mobile phase B for the first 6 min, gradually increased to 35% mobile phase B over the next 80 min, then increased to 85% mobile phase B over 5 min with a 5 min hold at a constant flow rate of 0.3 µL/min.

### Proteomics data analysis

For every LC–MS run, the raw data file obtained was subjected to two searches using Byonic 2.11.0 (Protein Metrics, San Carlos, CA). The first search was performed against a Swiss-Prot database containing the reference human proteome (2022; 20,645 entries), while the second search was conducted against a Swiss-Prot database containing the reference mouse proteome (2022; 17,380 entries). The search parameters for the database included trypsin digestion with a maximum of two missed cleavages, a precursor mass tolerance of 0.5 Da, and a fragment mass tolerance of 10 ppm. Additionally, fixed cysteine carbamidomethylation was specified, along with variable modifications such as methionine oxidation and asparagine deamination. To ensure high confidence in the peptide identifications, peptides with a false discovery rate (FDR) exceeding 1% were filtered out. Furthermore, peptides identified in both the human and mouse database searches were eliminated, enabling a conservative analysis of human-identified proteins with non-homologous mouse peptides. This analysis was performed using an in-house R script, considering three biological replicates per cell line (control-GFP and AZGP1-OV PC3) and three technical replicates for each experimental condition. Protein levels were determined by examining the signals specific to each protein within the samples. To determine the relative abundance of proteins, these signals were compared to the average signal observed across all samples. This enabled the evaluation of each protein's abundance relative to the overall protein levels in the experimental dataset. To ensure comparability and facilitate statistical analysis, a normalization procedure was applied. The relative abundance values were adjusted to a normal distribution with a mean of 0 and a standard deviation of 1. Finally, statistical analysis involved the application of the Student's T-test for comparing pairs and Pearson’s correlation test for comparing the cell lines. For further analysis, only proteins demonstrating a significance level below 0.01 were included in the subsequent analyses.

To assess the fold change and significance of proteins, we employed uncorrected t-test comparing control GFP PC3 cells (N = 9 biological replicates) with AZGP1-OV PC3 tumors (N = 9). For statistical analysis, we conducted a multiple hypothesis correction separately for all data (gray) and exclusively for genes associated with angiogenesis (GO:0001525). To perform this correction, we utilized the Benjamini–Hochberg procedure with a false-discovery rate of 10% (FDR = 0.1) as described previously [26]. Heatmaps were generated by clustering genes hierarchically/agglomeratively using the 'average' method and 'euclidean' metric. For the enrichment analysis of genes involved in angiogenesis in control GFP and AZGP1-OV PC3 tumors, we employed a null hypothesis assuming that these genes were randomly selected from the distribution of proteins confidently measured in mass spectrometry. Out of the 2803 proteins with sufficient signal-to-noise for quantification, 94 were identified as angiogenesis-related, while 1282 exhibited significant differences in protein expression (above the Benjamini–Hochberg multiple hypothesis cutoff). To model the count distribution, we employed a hypergeometric distribution (N = 2803, n = 1282, K = 94).

### Transcriptomic data analysis

All transcriptome data were obtained from cBioPortal for Cancer Genomics (https://www.cbioportal.org/). The mRNA levels of AZGP1 in PCa and patient data were collected from the MSKCC Prostate Cancer Genomics Data Portal (http://cbio.mskcc.org/prostate-portal/) for the MSKCC-PRAD dataset [27] and the Genomic Data Commons Data Portal (https://portal.gdc.cancer.gov) for the TCGA-PRAD dataset. The associations of AZGP1 with disease-free survival and Gleason scores were determined by Python.

We identified transcripts correlated with AZGP1 gene expression using the bulk RNAseq data from the TCGA PRAD dataset. To measure correlations, we employed Kendall's tau coefficient, a non-parametric rank correlation test that is sensitive to non-linear relationships [28]. We assessed significance and controlled for false discoveries by shuffling AZGP1 expression values across samples and recalculating Kendall's tau across the entire dataset. We determined significance for both positive and negative correlations separately. All analysis is performed in Python 3.7.4 with the modules numpy (1.18.1), scipy (1.4.1), and pandas (1.2.3). Kendall's tau was calculated with method set to 'kendall' in pandas.DataFrame.corrwith. The angiogenesis related genes were obtained from GO:0001525. The Gene Ontology (GO) enrichment analysis and WikiPathways analysis was performed using g: Profiler tool[29].

### Patient

The study was performed with guidelines provided by Institutional Review Board at Stanford University (IRB: 11612). All patients (n = 215) underwent prostate cancer surgery in1985-1997. Patient’s with postoperative mortality < 30 days, follow-up of < 3 years without recurrence, simultaneous with other types of cancers were excluded from the study.

### Tumor tissue microarray staining and analysis

To determine the relationship between AZGP1 expression and microvessel density in human prostate cancers, we used a tissue microarray comprised of 215 patient samples from radical prostatectomies performed in men with clinically localized prostate cancer. The microarrays include up to four cores (0.6mm) from each case. The TMAs were stained for multiplexed immunofluorescence (MxIF) imaging as previously described[30]. Briefly, multiplexed immunofluorescence was performed using a Cell DIVE imager (Leica Microsystems, Issaquah, WA) using a 20 × 0.75 NA objective. AZGP1 expression was captured by measuring quantitative fluorescence in the cancer epithelial cell compartment that had been segmented by methods described previously[31, 32]. Blood vessels were automatically segmented based on a combination of immunofluorescent staining for CD31, CD34 and collagen IV, using a previously published deep learning framework[30]. Subsequently, the microvessel density was calculated as the number of segmented microvessels per core. Microvessel density was correlated with AZGP1 protein expression by averaging log2 transformed intensity in epithelial cells in each core. Correlation was measured using Pearson correlation on a continuous scale, and we also divided cores into two groups using median microvessel density, then compared AZGP1 intensity between the two groups (low/high) using the Wilcoxon test.

### Statistical analysis

Statistical analysis of the experimental data was performed using GraphPad Prism software (StataCorp., College Station, TX). All data are presented as mean ± standard error of the mean (S.E.M). For comparisons between two groups, an unpaired Student's t-test was used. When comparing more than two groups, a one-way analysis of variance (ANOVA) followed by the Tukey’s test for post hoc analysis was applied to determine significant differences. ns: non-significant p > 0.05, *p < 0.05, **p < 0.01, ***p < 0.001, ****P < 0.0001.

## Results

### *AZGP1 does not affect cell proliferation in PCa *in vitro*.*

**Fig. 1:**
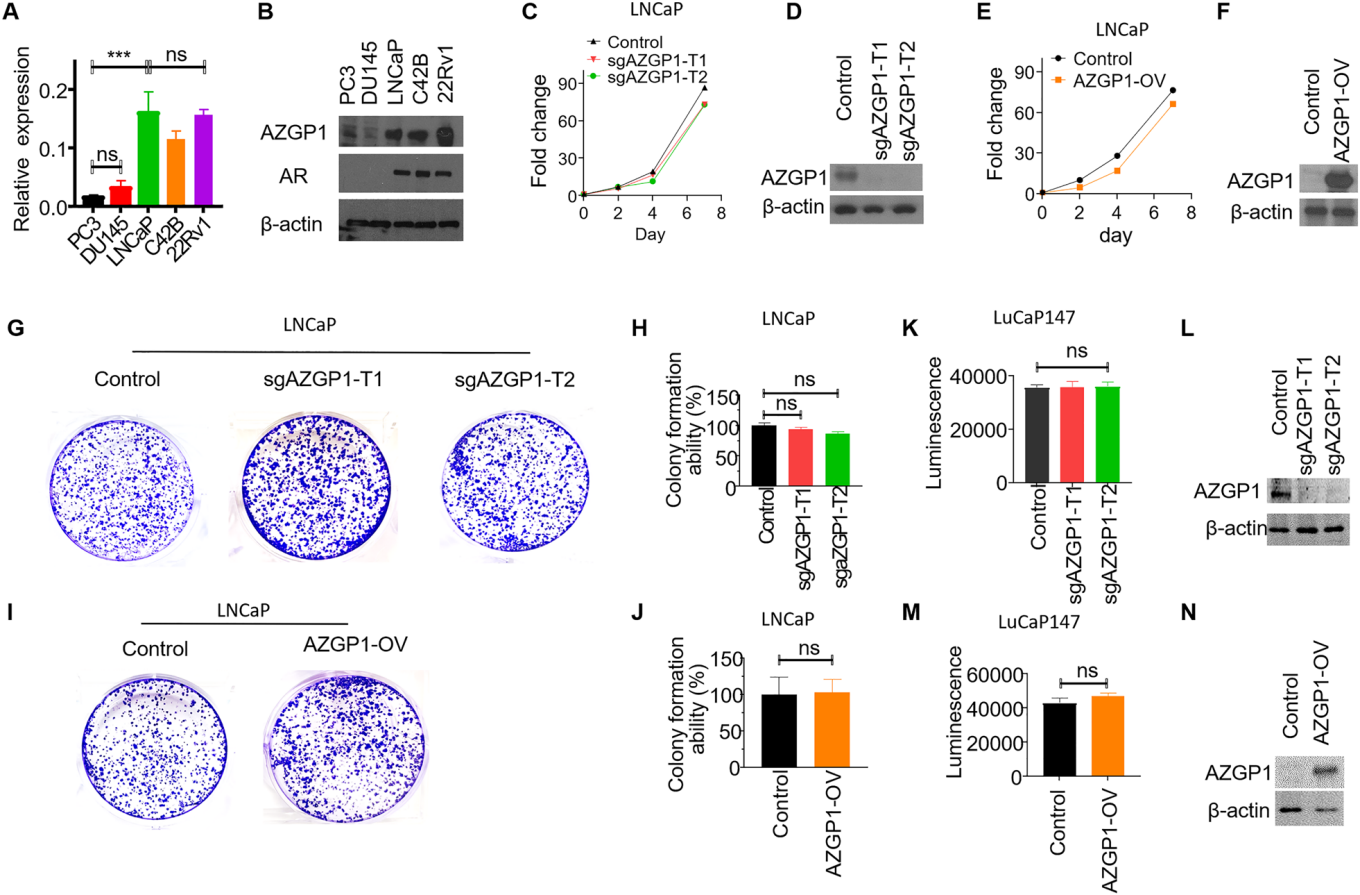
AZGP1 has no impact on cell proliferation in PCa cells. **A** Transcript expression levels of AZGP1 are higher in LNCaP, C42B, and 22Rv1 cell lines compared to PC3 and DU145 cells, as determined by RT-qPCR. **B** Western blot analysis of protein levels of AZGP1 are higher in LNCaP, C42B, and 22Rv1 cell lines compared to PC3 and DU145 cells. **C** Knockout of AZGP1 in LNCaP sg-AZGP1-T1 and sg-AZGP1-T2 cells showed no significant effect on cell proliferation. **D** Western blot confirming the knockout of AZGP1 in LNCaP sg-AZGP1-T1 and sg-AZGP1-T2 cells. Blot membrane was incubated with secondary antibody overnight. **E** Overexpression of AZGP1 in LNCaP AZGP1-OV cells did not impact cell proliferation. **F** Western blot confirming increased AZGP1 protein expression in LNCaP AZGP1-OV cells. The blot membrane was incubated with secondary antibody for 1 h. **G** Knockout of AZGP1 had no effect on colony formation in LNCaP cells. **H** Quantification of colony formation in control and AZGP1 knockout sg-AZGP1-T1 and sg-AZGP1-T2 LNCaP cells. **I** Overexpression of AZGP1 had no effect on colony formation in LNCaP cells. **J** Quantification of colony formation in LNCaP control and AZGP1-OV cells. **K** Knockout of AZGP1 had no significant effect on cell proliferation in LuCaP147 cells. **L** Western blot confirming the knockout of AZGP1 in LuCaP147 sg-AZGP1-T1 and sg-AZGP1-T2 cells. Blot membrane was incubated with secondary antibody overnight. **M** Overexpression of AZGP1 did not affect cell proliferation in LuCaP147 cells. **N** Western blot confirming the overexpression of AZGP1 in LuCaP147 AZGP1-OV cells. Blot membrane was incubated with secondary antibody for 1 h. Note: the exposure time for AZPG1 knockout cells (LNCaP control, sg-AZGP1-T1 and sg-AZGP1-T2 cells, LuCaP147 control, sg-AZGP1-T1 and sg-AZGP1-T2 cells,) and AZGP1 overexpression cells (LNCaP control and AZGP1-OV, LuCaP147 control and AZGP1-OV cells) are different

Loss of AZGP1 protein and RNA expression has been associated with adverse outcomes in PCa in multiple datasets. To investigate the functional role of AZGP1, we determined the expression levels of AZGP1 in various PCa cell lines. Consistent with previous findings [21], both transcript and protein levels of AZGP1 were much higher in androgen receptor (AR)-positive cell lines (LNCaP, C42B and 22Rv1), compared to the AR-negative cell lines DU145 and PC3, determined by qPCR and western blot, respectively (Fig. 1A, B).

Knockout of AZGP1 in LNCaP cells did not change proliferation rates compared to wild-type LNCaP cells that expressed AZGP1 (Fig. 1C), suggesting AZGP1 is not required for LNCaP proliferation in vitro. The absence of expression of AZGP1 protein in the knockout cells was demonstrated by western blot (Fig. 1D). Interestingly, overexpression of AZGP1 in LNCaP cells also did not alter their rate of proliferation (Fig. 1E, F). Moreover, colony formation assays using LNCaP cells, LNCaP AZGP1-knockout or LNCaP cell overexpressing AZGP1 showed that changes in AZGP1 level did not affect LNCaP colony formation r growth (Fig. 1G–J). Similarly, overexpression of AZGP1 did not affect 22Rv1 cell proliferation and colony formation ability (Additional file 1: Fig S1A–D).

Patient Derived Xenografts (PDXs) have demonstrated the ability to faithfully replicate the histological and molecular characteristics of the original tumors, including responses to standard-of-care therapies [33]. The LuCaP147 PDX serves as an authentic model of human PCa subtype with an SPOP mutation and a hypermutator phenotype [22, 23]. We evaluated the effect of AZGP1 on cell proliferation in LuCaP147 spheroids derived from a PDX tumor. LuCaP147 cells express moderate levels of AZGP1, and we therefore generated AZGP1 knockdown and over-expressing LuCaP147 cell lines and confirmed AZGP1 expression levels by qPCR and western blot (Fig. 1K, L). There was no significant impact of knockout or overexpression of AZGP1 on the proliferation of LuCaP147 cells (Fig. 1K–N). Therefore, in contrast to previous reports [21], we found that AZGP1 had no cell autonomous effects on PCa cell proliferation. These results suggest that the negative association of AZGP1 with survival is mediated by mechanisms other than inhibition of PCa proliferation.

### *AZGP1 does not affect PCa cell migration and invasion *in vitro*.*

**Fig. 2:**
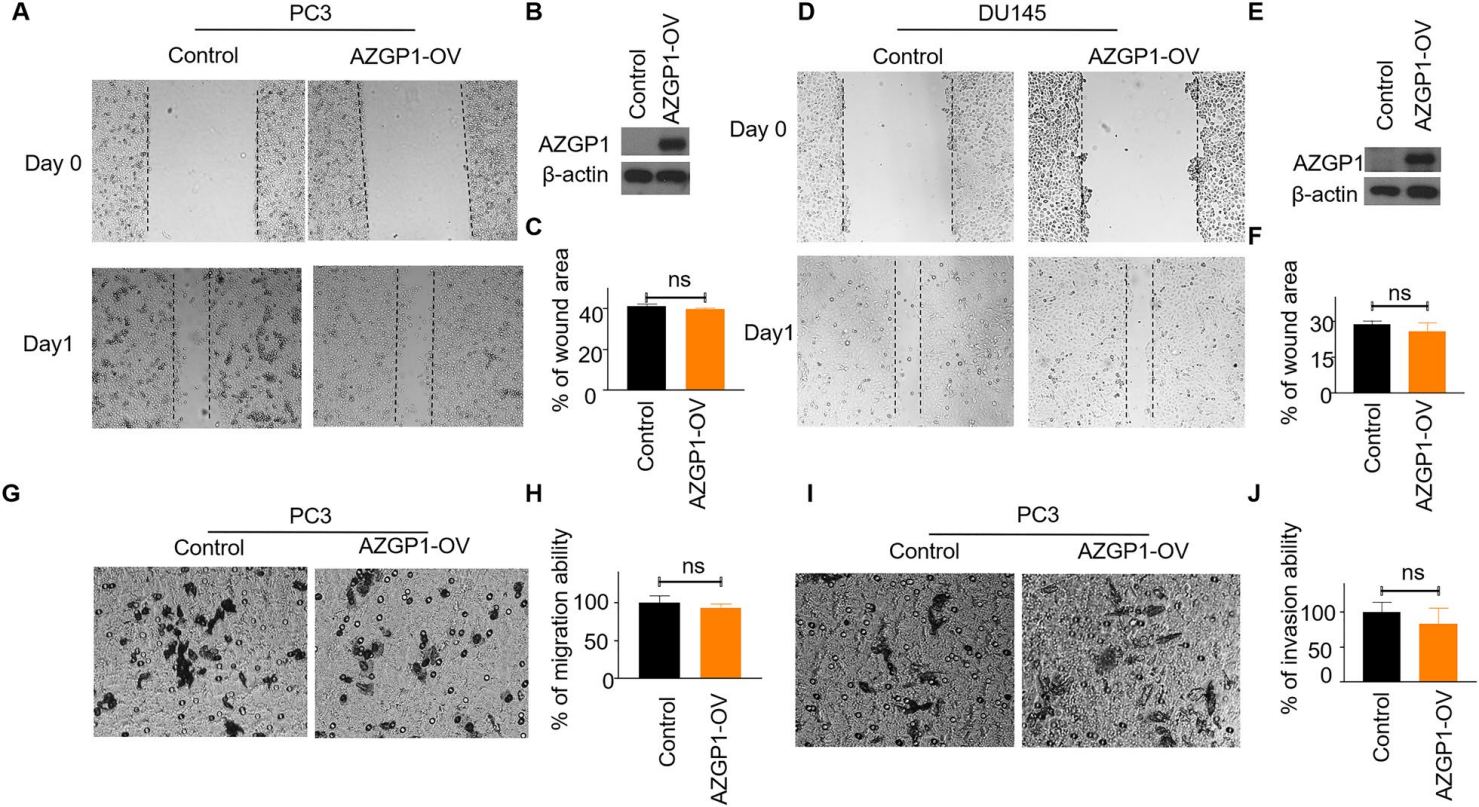
AZGP1 does not affect cell migration or invasion in PCa cells. **A** Wound-healing assay of PC3 vector control and PC3 AZGP1-OV cells shows no effect on cell migration. **C** Quantification of migration in PC3 vector control and PC3 AZGP1-OV cells. **B** Western blot confirming the overexpression of AZGP1 in PC3 AZGP1-OV cells. **D** Wound-healing assay of DU145 vector control and DU145 AZGP1-OV demonstrating no effect of AZGOP1 on DU145 cell migration. **F** Quantification of migration ability in DU145 vector control and DU145 AZGP1-OV cells. **E** Western blot confirming the overexpression of AZGP1 in DU145 AZGP1-OV cells. **G** Transwell migration assay PC3 vector control and PC3 AZGP1-OV demonstrating does not affect PC3 cell migration. **H** Quantification of migration ability in PC3 vector control and PC3 AZGP1-OV cells. **I** Transwell invasion assay of PC3 vector control and DU145 AZGP1-OV showing that overexpression of AZGP1 does not affect PC3 cell invasion. **J** Quantification of invasion ability in PC3 control and AZGP1-OV cells

We tested the role of AZGP1 in PCa cell migration and invasion using PC3 and DU145 cell lines since they are highly invasive and migratory. Wound-healing assays demonstrated that overexpression of AZGP1 did not alter cell migration in either PC3 (Fig. 2A–C) or DU145 cells (Fig. 2D–F). Likewise, transwell migration and invasion assays showed that overexpression of AZGP1 had no significant effect on PC3 migration (Fig. 2G, H) and invasion (Fig. 2I, J). Therefore, we observed no cell-autonomous effects of AZGP1 expression on cell migration or invasion in PCa cells, as had been reported previously [21].

### ***AZGP1***^−***/***−^*** mice show heterogeneous changes in mesenchymal composition***

**Fig. 3:**
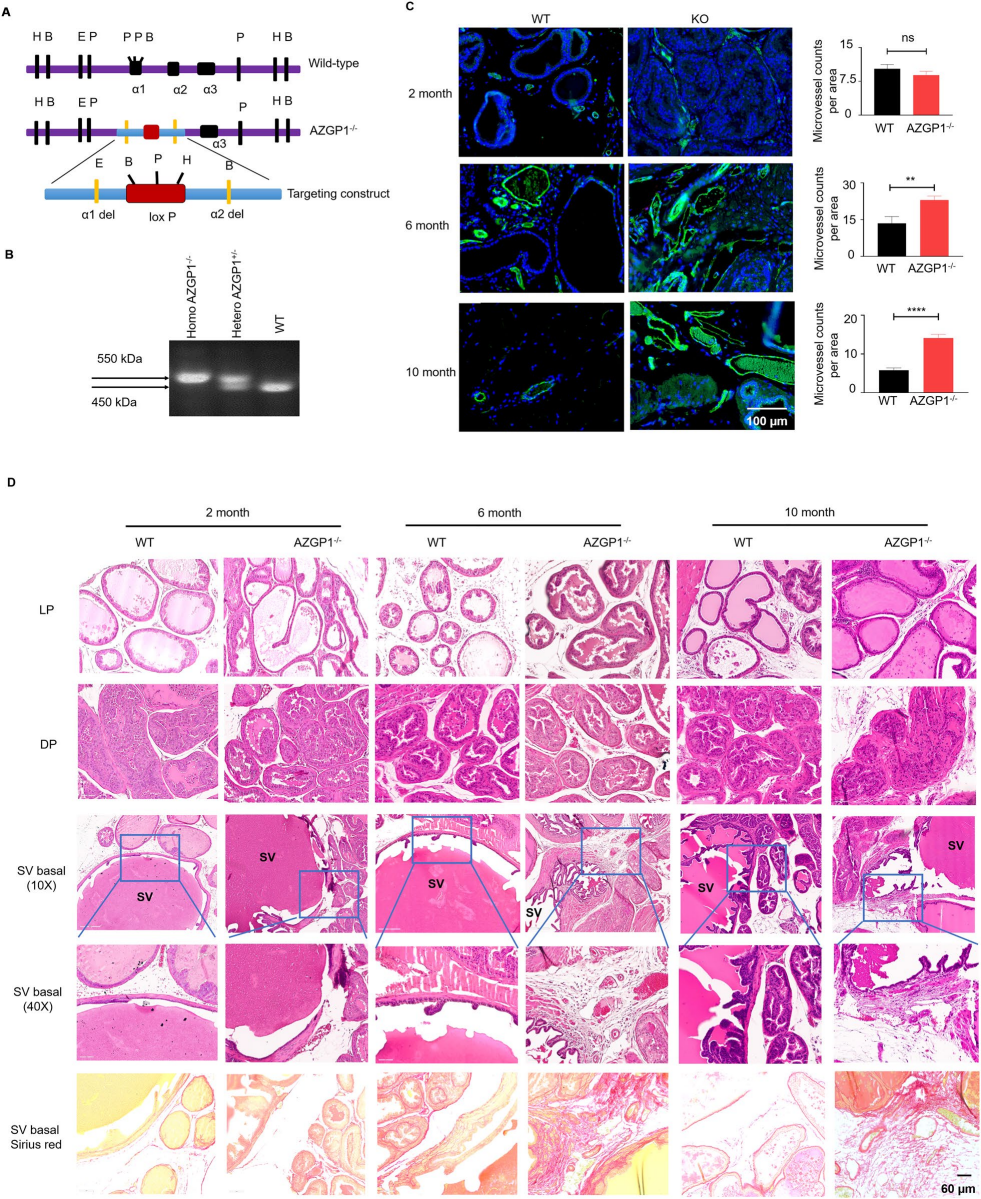
Loss of AZGP1 promotes stromal cell hyperplasia in the prostate of AZGP1^−/−^ mice. **A** Schematic representation of endogenous AZGP1, AZGP1 knockout, and targeting construct. **B** Genotype analysis of AZGP1 of wild-type, heterozygous AZGP1^±^ and homozygous AZGP1^−/−^ mice. **C** Immunofluorescence staining of CD31 and its corresponding quantification of microvessle counts per area in prostate excluding SV from wild-type and homozygous AZGP1^−/−^ mice. Increased microvessel density was observed in homozygous AZGP1^−/−^ mouse prostates. Microvessels were counted from 10 randomly selected 20X microscope fields in the CD31-stained sections. **D** Hematoxylin and eosin (H&E) staining of the *LP* lateral prostate, *DP* dorsal prostate and basal part of SV seminal vesicles from wild-type and homozygous AZGP1^−/−^ mice suggest that there are no morphological changes in LP and DP. Increased fibrosis were observed at the base of the seminal vesicles as illustrated with sirius red staining

Although AZGP1 loss is associated with adverse clinical outcomes, unexpectedly, we did not observe a direct effect of AZGP1 on the proliferation, clonal growth, migration, or invasion of PCa cell lines. Therefore, we used a mouse model to examine the role of AZGP1 in the prostate in vivo*,* where the microenvironment is intact. AZGP1^−/−^ mice were bred and genotyped using a targeting construct that specifically deleted the α1 and α2 domains of AZGP1 (Fig. 3A) [25] which produces a modified non-functional 550 kDa allele, compared to the wild-type allele of 450 kDa (Fig. 3B).

Since AZGP1 has never been assessed in normal prostate biology, we first examined the histology of prostates from AZGP1^−/−^ mice. Prostates from male mice were harvested at 2 months, 6 months and 10 months of age. Though there was a mild phenotype in 2 month-old prostates, significantly increased blood vessel density was observed in the mesenchyme of the prostates from 6 month-old to 10 month-old AZGP1^−/−^ mice (Fig. 3C). This phenotype was present in the proximal and distal regions of several lobes, though it was enriched in the proximal and periurethral region of the lobes. There were no significant differences in the epithelial anatomy of the dorsal and lateral prostate lobes between wild-type and AZGP1^−/−^ mice. However, increased fibrosis and collagen deposition could be observed, which was most apparent proximally and near the base of the seminal vesicles. Similar to the endothelial phenotype, this was seen in the prostates of 6-month-old and 10-month-old AZGP1^−/−^ mice, as confirmed by Sirius red staining (Fig. 3D).

### AZGP1 inhibits angiogenesis in PCa xenografts

**Fig. 4:**
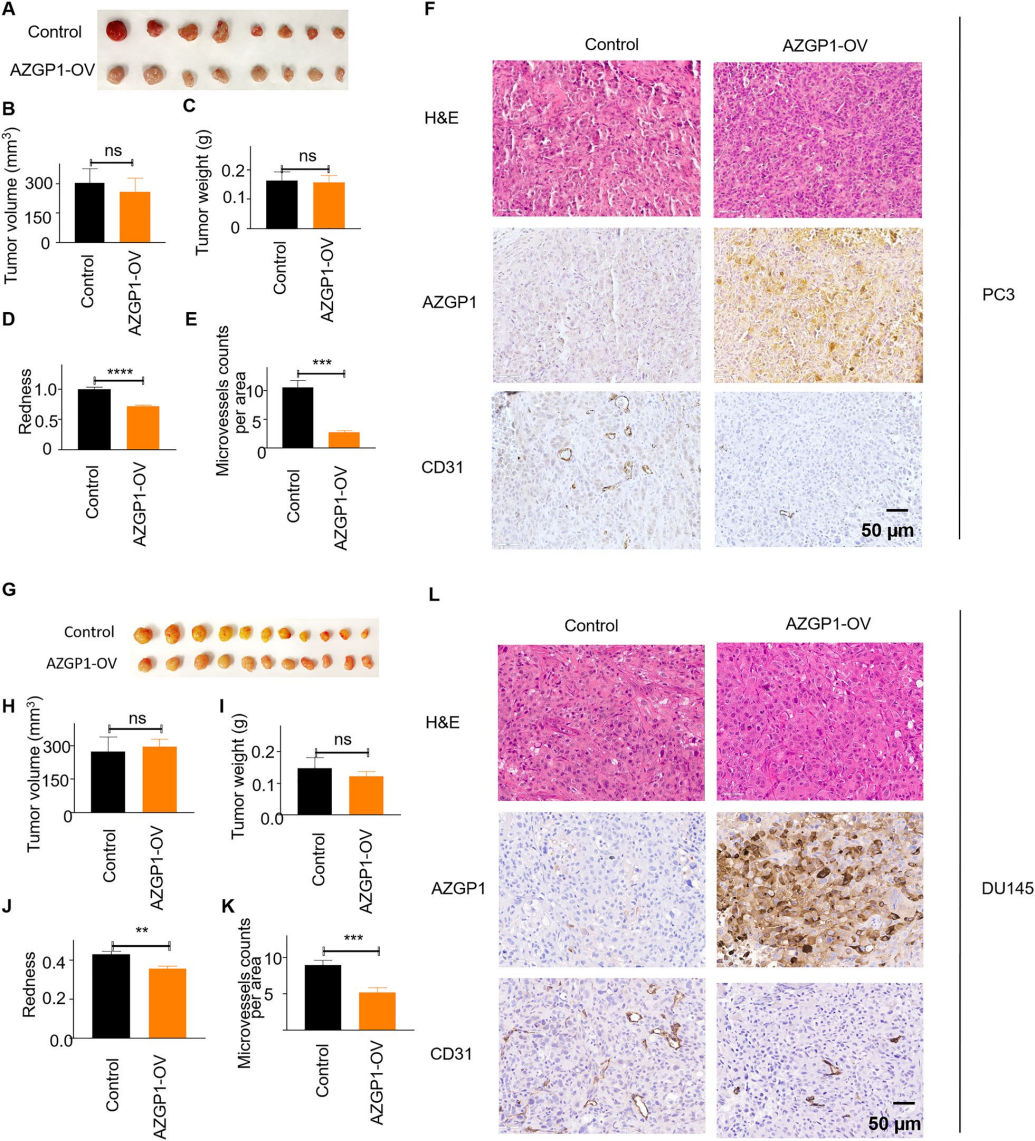
Overexpression of AZGP1 inhibits angiogenesis in PC3 and DU145 tumors. **A** Representative images of PC3 vector control and PC3-AZGP1-OV tumor xenografts. **B** Calculated tumor volume, and **C** Tumor weight of PC3 vector control and PC3-AZGP1-OV tumor xenografts. **D** Quantification of the degree of redness of PC3 vector control and PC3-AZGP1-OV tumor xenografts using python, showing reduced redness in AZGP1-OV PC3 tumors. **E** CD31 staining showing lower microvessel density in AZGP1-OV PC3 tumors compared to controls. Microvessels were counted from 10 randomly selected high powered microscope fields uon the CD31-stained sections. **F** Microscopic images showing similar tumor morphology by H & E, validation of AZGP1 overexpression, and decreased number of CD31 staining microvessels in PC3-AZGP1-OV tumors compared to controls. **G** Representative images of DU145 vector control and DU145-AZGP1-OV tumor xenografts. **H** Calculated tumor volume, and **I** tumor weight of control and AZGP1-OV DU145 tumors. **J** Quantification of tumor redness using Image J, indicating reduced redness in AZGP1-OV DU145 tumors compared to vector control. **K** DU145 AZGP1-OV tumors displayed lower microvessel density compared to controls. **L** Microscopic images showing similar tumor morphology by **H** and **E**, validation of AZGP1 overexpression, and decreased number of CD31 staining microvessels in DU145-AZGP1-OV tumors compared to controls

While in vitro experiments suggested no effects of AZGP1 on cancer cell growth, AZGP1 knockout mice showed changes in the prostate stroma, and we tested whether altering expression of AZGP1 would affect PCa growth in vivo. Control PC3 and AZGP1-OV PC3 cells were implanted into the dorsal flank of 10–12 week-old NSG mice and propagated for 41 days. Similar to our observations in vitro, we found no change in tumor size, weight or volume in AZGP1-OV PC3 group compared to controls (Fig. 4A–C). However, we noticed that the PC3-AZGP1-OV tumors were much more pallid compared to the PC3 control tumors. We quantified the degree of redness in the PC3 control tumors compared to the PC3-AZGP1-OV tumors and confirmed that there was a significantly greater red-hued pixels in the low-AZGP1-expressing controls compared to the AZGP1 over-expressing xenografts (Fig. 4D). We reasoned that this difference was due to an increased number of small vessels per area, referred to as microvessel density, in the tumors. Immunohistochemistry confirmed increased expression of AZGP1 in the PC3-AZGP1-OV compared to controls (Fig. 4F). Interestingly, tumors expressing high levels of AZGP1 showed significantly lower microvessel density compared to the low-AZGP1 expressing controls, quantified by IHC using CD31 antibody and manual counting of vessels in 40X microscopy fields (Fig. 4E, F). These findings were also validated in xenografts of DU145 tumors that natively express low levels of AZGP1 and DU145 cells overexpressing AZGP1. Once again, over-expression of AZGP1 did not affect tumor size, volume or weight, but did decrease tumor redness and microvessel density compared to controls, albeit to a slightly lesser degree compared to PC3 cells (Fig. 4G–L). Similarly, over-expression of AZGP1 did not affect tumor growth for AR-positive 22Rv1 cells (Additional file 1: Fig. S1E, F). We did not observe any changes in tumor growth in AZGP-OV 22Rv1 tumors compared to control 22Rv1 tumors. AZGP1 did not affect microvessel denisty in 22Rv1 tumors (Additional file 1: Figure S1G-H), although 22Rv1 cells express high levels of AZGP1 at baseline. Together these results suggest that AZGP1 does not affect growth in vivo; however, loss of AZGP1 expression appears to promote increased microvessel density and tumor vascularization.

### AZGP1 regulates expression of proteins involved in angiogenesis in PCa xenografts

**Fig. 5:**
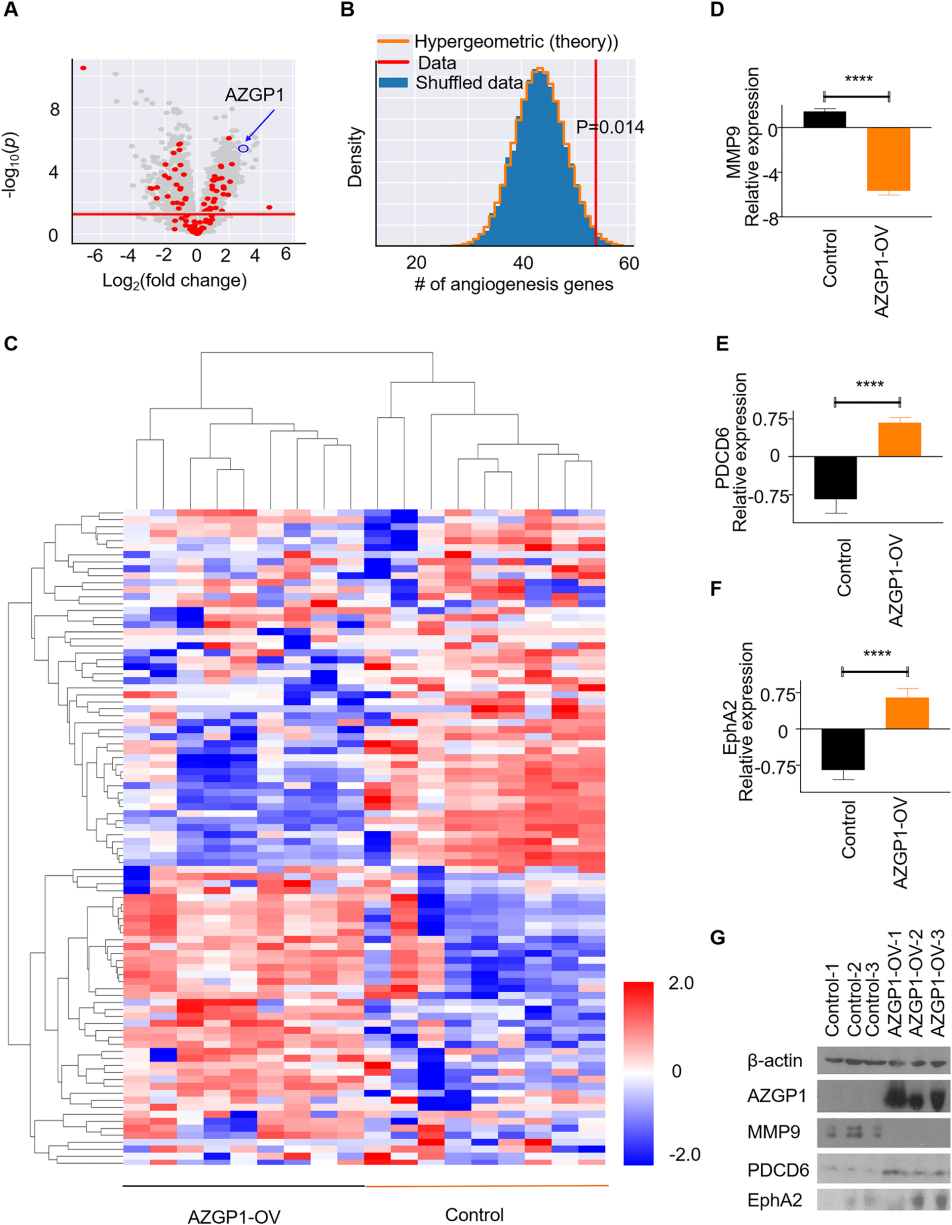
AZGP1 protein expression in PC3 cell lines modulates the angiogenesis pathway. **A** Volcano plot of proteome of PC3 vector control and PC3-AZGP1-OV shows 1282 significantly modulated proteins (grey dots) and 94 were identified as angiogenesis-related genes (red dots). Proteins illustrated in red demonstrate 29 upregulated genes and 25 downregulated genes that are associated with angiogenesis. **B** Hypergeometric distribution of angiogenesis genes shows that the identified 54 genes are significantly upregulated or downregulated between PC3 control and AZPG1-OV tumors (*p* = 0.014) **C** Heatmap of the 54 angiogenesis genes in AZGP1-OV PC3 and controls shows clear segregation of the over-expressing and low expressing cells. **D** Relative protein expression levels of MMP9, **E** PDCD6, **F** EphA2 in AZGP1-OV PC3 and control tumors. **G** Western blot confirming decreased levels of MMP9 and increased levels of PDCD6 and EphA2 proteins, and validation of AZGP1 overexpression in AZGP1-OV PC3 tumors

We performed mass spectrometry-based proteomic analysis using tumor tissues from the control and AZGP1-OV PC3 groups. The volcano plot in Fig. 5A demonstrated high expression of AZGP1 protein in the PC3-AZGP1-OV cells, as expected, as well as proteins significantly upregulated and down-regulated in response to AZGP1 overexpression. We cross-referenced the differentially expressed proteins in control PC3 and PC3-AZGP1-OV tumors and found significant overlap of proteins associated with the angiogenesis pathway (GO:0001525) (Fig. 5A and B, *p* = 0.014 by hypergeometric distribution). Hierarchical clustering of tumors using the proteins identified in the angiogenesis pathway demonstrated clear segregation of AZGP1 overexpressing tumors from controls for both up- and down-regulated proteins (Fig. 5C). Of the differentially expressed genes, 29 genes related to angiogenesis were downregulated and 25 genes were upregulated in the AZGP1-OV tumors compared to the PC3 control group tumors (Additional file 1: Table 1). Among the top differentially expressed angiogenesis-related proteins, MMP9, GPX1, HSPB1, COL18A1, RNH1, and ANXA1 showed negative correlation with AZGP1 overexpression (Fig. 5D, and Additional file 1: Fig S2A–E), while SERPINE1, YWHAZ, EphA2, and PDCD6 exhibited positive correlation with AZGP1 overexpression (Fig. 5E, F, and Additional file 1: Fig S2F–G). Downregulation of MMP-9 and upregulation of PDCD6 and EphA2 in AZGP-OV tumors were confirmed by Western blot (Fig. 5G). Proteomic analysis of DU145 showed similar trends of these angiogenesis-related proteins, although several were not stastically significant likely due to the subtler differences in microvessel density in DU145 (Additional file 1: Fig S3). These results suggest that AZGP1 inhibits angiogenesis in PCa through regulating known angiogenesis associated proteins.

### AZGP1 inhibits HUVEC proliferation, migration and tube formation

**Fig. 6:**
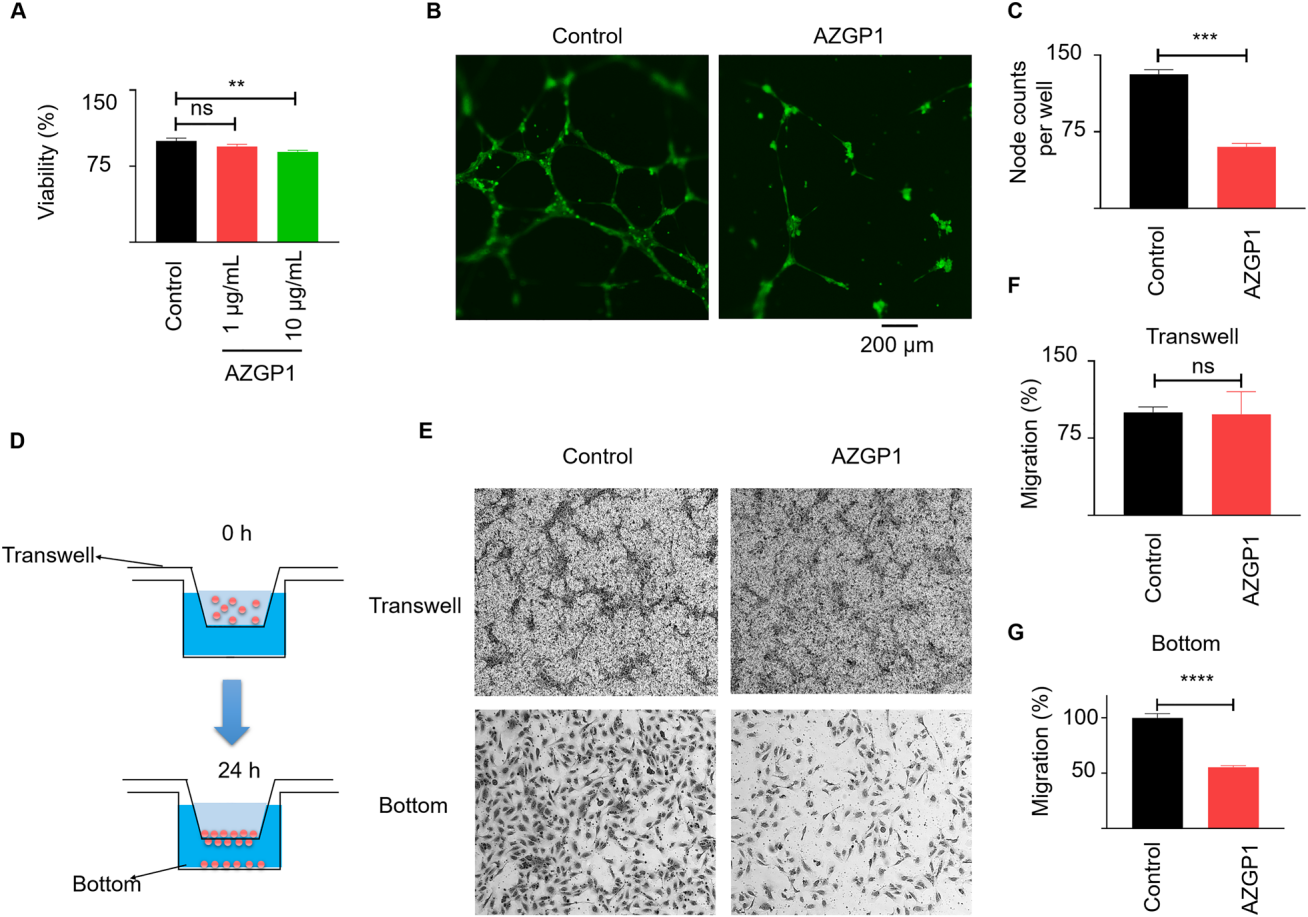
AZGP1 inhibits HUVEC proliferation, migration and tube formation. **A** AZGP1 slightly inhibits the HUVEC growth after 7-days treatment. **B** AZGP1 treatment inhibits tube formation ability and node formation of HUVECs. **C** Quantification of HUVEC nodes counted from 10 hpf. **D** Diagram summarizing the transwell migration assay. **E** There were no differences in cells migrating to the bottom of the transwells in AZGP1-treated and untreated HUVECs. However Significantly fewer cells were found in the bottom wells in AZGP1-treated HUVECs. The corresponding quantification of cells being migrated to (**F**) bottom of the transwell and, **G** the bottom of the wells

To test directly the effects of AZGP1 on angiogenesis, we performed several angiogenesis assays using HUVECs in vitro. After 7 days, AZGP1 inhibited the HUVEC growth, albeit to a small, but significant, degree (Fig. 6A). AZGP1 treatment effectively suppressed HUVEC tube formation ability and node formation, as depicted in Fig. 6B, C. A Transwell migration assay showed comparable numbers of cells migrating to the bottom of the transwells for AZGP1-treated and untreated HUVECs. However, the AZGP1-treated group showed significantly fewer cells migrated to the bottom of the wells compared to the non-treated control HUVECs (Fig. 6D–G). These results suggest that AZGP1 inhibits proliferation, migration, and tube and node formation of HUVECs.

### AZGP1 expression is associated with an angiogenesis in human PCa tissues

**Fig. 7:**
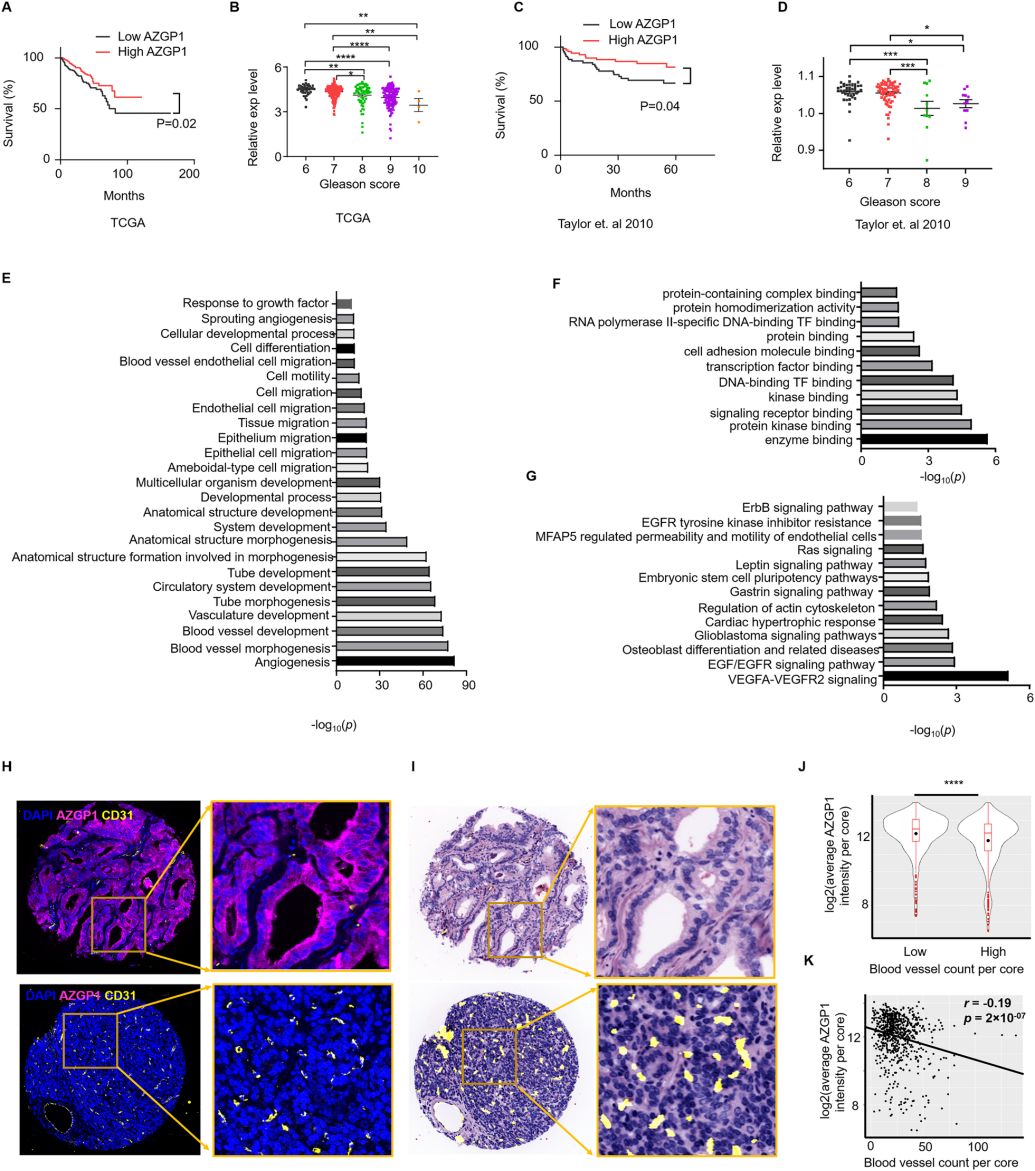
AZGP1 is involved in angiogenesis in human prostate cancer. **A** Low expression level of AZGP1 is correlated with poor survival in the PRAD-TCGA database. **B** Lower expression of AZGP1 is associated with higher Gleason scores in the PRAD-TCGA database. **C** Decreased expression level of AZGP1 shows strong correlation with poor survival in the MSKCC database. **D** Lower expression of AZGP1 is associated with higher Gleason scores in the MSKCC database. **E** GO biological process analysis of the 74 overlapping genes shows significant roles in angiogenesis. **F** GO molecular function analysis of the 74 overlapping genes. TF: transcription factor **G** WikiPathways analysis of the 74 genes implicates the VEGF signaling pathway as the possible target of AZGP1 expression modulation in prostate cancer cells. **H** Representative images of immunofluorescence staining of DAPI, AZGP1, and CD31 in a human tissue microarray (TMA) of localized prostate cancer. **I** Representative image of a virtual hematoxylin and eosin stain of TMA cores to demonstrate the cancer morphology with superimposed microvessels determined by AI. **J** Human prostate tumor samples were divided at the median into high and low blood vessel density. AZGP1 expression was significantly higher in the low blood vessel density group compared to the high blood vessel density group. **K** The expression of AZGP1 is negatively correlated with blood vessel density

To further investigate the relationship of AZGP1 expression with angiogenesis, we interrogated existing PCa transcriptome databases. We first confirmed that AZGP1 levels in localized prostate cancers were associated with clinical outcome and Gleason score. In both TCGA-PRAD and MSKCC-PRAD, we divided samples at the median AZGP1 expression level into high and low expression cancers and found that low AZGP1 expression was significantly associated with poor disease-free survival outcomes (Fig. 7A, B), Additionally, low AZGP1 expression was associated with higher Gleason scores in both datasets (Fig. 7C, D).

With the knowledge that the transcript levels performed as expected in TCGA PRAD dataset, we then identified genes significantly associated with AZGP1 expression levels by correlation analysis with Kendall's tau. We performed Gene Ontology (GO) enrichment analysis to test whether AZGP1 expression in TCGA-PRAD is correlated with angiogenesis-associated transcripts in human PCa. By comparing transcript levels in the GO angiogenesis pathway with AZGP1-associated transcripts, we identified 74 common transcripts that were present in both gene lists (Fig. 7A). The GO term analysis of the biological function of these 74 genes revealed enrichment in blood vessel morphogenesis, blood vessel development, vasculature development, tube morphogenesis and development, endothelial and epithelial cell migration, and response to growth factors (Additional file 1: Fig. S4). The molecular functions enriched included genes associated with enzyme binding, protein kinase binding, signal receptor binding, and transcription factor binding (Fig. 7E). Furthermore, a WikiPathways analysis demonstrated that the 74 genes were enriched several signaling pathways including VEGFA-VEGFR2, EGF/EGFR, Ras, EGFR, and ErbB (Fig. 7F). Together, these results suggest that AZGP1 works in part through constraining cancer progression though inhibiting angiogenesis in human PCa.

We had noted above that the angiogenesis phenotype was found predominantly in AR negative PCa xenografts. To evaluate whether AZGP1 could affect microvessel density in AR + clinically localized human prostate cancer, we used the CellDIVE platform to perform multiplex immunohistochemistry analyses on a human prostate cancer tissue microarray (TMA) containing 215 cases. We have developed a deep-learning model to identify blood vessels based by combining CD31, CD34 and collagen IV images and tabulated vessel numbers per core to capture microvessels[30]. Representative staining of high AZGP1 expression, captured based on quantitative fluorescence and low numbers of microvessels (Fig. 7H) and low AZGP1 expression and high microvessel numbers (Fig. 7I) were captured. Based on previous studies that manually score AZGP1 protein expression into high and low categories, when we dichotomized our samples into high and low expression based on the median, we found that AZGP1 expression was inversely correlated with microvessel numbers in the cores (*P* < 0.0001, Fig. 7J). Analyzing AZGP1 expression as a continuous variable showed a modest, but highly significant inverse correlation between AZGP1 expression and microvessel number (*r* = −0.19, *p* = 2 × 10^−7^, Fig. 7K).

## Discussion

Modulation of AZGP1 expression in vivo results in changes in the stromal compartment of normal and malignant prostate tissues. In the AZGP1 knockout mouse, loss of expression is associated pergladular fibrosis over time as well as increased vascularity. In PCa cell lines that express little AZGP1 protein at baseline, over-expression of AZGP1 significantly decreases microvessel density in mouse xenograft models. These findings are consistent with clinical data demonstrating that AZGP1 loss is strongly associated with adverse outcomes in PCa and suggests that loss of expression promotes tumor angiogenesis. Furthermore, the inverse correlation of AZGP1 expression and microvessel density observed in localized human prostate cancers confirms the inhibitory effect observed in the animal models. In agreement with this hypothesis, low AZGP1 expression level in PC3 and DU145 tumor xenografts is associated with increased expression of angiogenic proteins. In addition, in TCGA-PRAD, genes associated with AZGP1 expression overlap with many genes associated with angiogenesis, implying this observation is relevant to human early staged PCa.

Angiogenesis, the process of forming new blood vessels, is crucial for various physiological and pathological processes, including tumor growth and metastasis. It involves the migration, proliferation, and differentiation of endothelial cells to form new blood vessels and supports tumor growth and metastasis by facilitating the delivery of oxygen and nutrients to the growing tumor [35]. Many reports demonstrate that increased microvessel density in PCa is associated with adverse outcomes [36–39]. Proteomic analysis of AZGP1 overexpressing cells showed repression of MMP9, GPX1, HSPB1, COL18A1, RNH1, and ANXA1, as well as increased expression of SERPINE1, YWHAZ, EPHA2, and PDCD6, and all of these proteins have been implicated in angiogenesis. For example, MMP-9 causes release of vascular endothelial growth factor (VEGF) to promote angiogenesis [40], and inhibition of MMP-9 decreases both cell migration and angiogenesis in retinoblastoma. [41]. GPX1-deficient mice display impaired angiogenesis, likely due to the inability of endothelial progenitor cells to stimulate angiogenesis because of elevated levels of reactive oxygen species (ROS) [42]. ANXA1 has been implicated in vascular endothelial cell sprouting, and ANXA1 inactivation has been shown to impair angiogenesis, tumor growth and metastasis in mouse models [43]. ANXA1 has been demonstrated to promote endothelial tube formation and enhance VEGF secretion by activating the formyl peptide receptor FPR2, and increased levels of ANXA1 correlate with angiogenesis in breast cancer [44]. On the other hand, PDCD6 shows increased expression in AZGP1 over-expression, and has been reported to exert a suppressive effect on angiogenesis by binding to VEGFR-2, leading to the phosphorylation of PI3K and subsequent regulation of downstream signaling regulators. Overexpression of PDCD6 has shown to significantly inhibit VEGF-induced migration and tube formation of HUVECs[45]. Examination of transcripts correlated with AZGP1 expression in TCGA were enriched for key biological processes related to blood vessel development, vasculature formation, tube morphogenesis, and migration of endothelial and epithelial cells, confirming the association of AZGP1 with angiogenesis in clinical samples. We have validated those findings in HUVECs, showing that administration of AZGP1 in the media inhibits HUVECs growth, migration, and tubular formation.

We found no evidence that AZGP1 affected cell growth, colony formation, migration or invasion, despite testing the several PCa cell lines, spheroids from the human patient derived xenograft LUCaP147, and numerous repeats of these experiments. Our findings contrast with the previous publication where AZGP1 over-expression was reported to increase cell proliferation and migration in an androgen receptor (AR)-dependent manner [21]. In addition, overexpression of AZGP1 did not have a significant impact on tumor growth of PC3, 22RV1, and DU145 xenograft models compared to vector controls with very low expression of AZGP1. Furthermore, our proteomic and transcriptomic analyses did not show a strong signal for pathways associated with cell proliferation, invasion, or metastases. In other words, we could find no evidence of cell autonomous effects of AZGP1 expression in PCa. Instead, both the AZGP1 knockout mice and the xenograft models point to heterotypic effects of AZGP1 on the prostate stromal microenvironment, where AZGP1 loss can promote proliferation of fibroblasts and angiogenesis.

The mechanisms underlying AZGP1’s effects on the prostate normal and tumor microenvironment are unclear. AZGP1 is a secreted glycoprotein [46] and could act as a ligand for specific receptors on fibroblasts or endothelial cells to directly affect angiogenesis and stromal cell growth [47]. For example, administration of exogenous AZGP1 to mice with ureteral obstruction decreases renal fibrosis, protecting against renal scarring and damage, implying it acts as a ligand in that it does not need to be expressed within a cell or tissue in order to show effects [48]. On the other hand, proteomics and transcriptomics of AZGP1-expressing and non-expressing cells showed alterations in several proteins implicated in angiogenesis and the extracellular matrix. One possible explanation of these findings is that AZGP1 acts indirectly in the cell by modulating effector proteins that mediate angiogenesis and stromal cell proliferation. AZGP1 has significant homology to the class I major histocompatibility complex (MHC) antigen-presenting proteins, leading to speculation that AZGP1 could also modulate the immune microenvironment [46]. However, since our in vivo experiments were conducted in immune-deficient NSG mice, we were unable to test whether AZGP1 modulates the immune cell microenvironment.

## Conclusions

We demonstrate that downregulation of AZGP1 does not impact cancer cell proliferation, migration, or invasion in PCa. Rather, AZGP1 loss appears to affect fibrosis and angiogenesis in the normal prostate in an AZGP1 knockout mouse model, and, importantly, angiogenesis in PCa cell line xenograft models and human surgical samples. Proteomics analysis of AZGP1 deficient and over-expressing PCa cell lines confirmed enrichment for pathways related to angiogenesis, including YWHAZ, EPHA2, SERPINE1, PDCD6, MMP9, GPX1, HSPB1, COL18A1, RNH1, and ANXA1. AZGP1 suppresses the growth, migration, and tube formation capacity of HUVEC cells. Our findings provide valuable insights into the anti-angiogenic properties of AZGP1 in PCa and suggests that AZGP1 could represent a therapeutic target in PCa. Further research is needed to elucidate the downstream signaling pathways involved in the anti-angiogenic function of AZGP1 for a comprehensive understanding of this important clinical biomarker of PCa aggressiveness.

### Supplementary Information

**Additional file 1: Figure S1.** (A) Overexpression of AZGP1 in AZGP1-OV 22Rv1 cells did not affect cell proliferation. (B) Western blot confirming increased AZGP1 protein expression in 22Rv1 AZGP1-OV cells. (C) Overexpressing of AZGP1 does not affect colony formation in 22Rv1 cells. (D) Quantification of colony formation in control and AZGP1-OV 22Rv1. (E) Rpresentative images of 22Rv1 vector control and 22Rv1 AZGP1-OV tumor xenografts. (F) Tumor weight of 22Rv1 vector control and 22Rv1 AZGP1-OV. (G) Representative images showing similar tumor morphology by H & E, validation of AZGP1 overexpression, and CD31 staining microvessels in 22Rv1-AZGP1-OV tumors and control tumors. **Figure S2.** Relative expression levels of (A) ANXA1, (B) HSPB1, (C) GPX1, (D) RNH1, (E) COL18A1, (F) YWHAZ, (G) SERPINE1 in PC3 control and AZGP1-OV tumors, determined by mass spectrometry. **Figure S3.** Relative expression levels of (A) GPX1, (B) RNH1, (C) COL18A1, (D) YWHAZ, (E) PDCD6 in DU145 control and AZGP1-OV tumors, determined by mass spectrometry. **Figure S4.** Genes correlated with AZGP1 expression levels in TCGA-PRAD overlapped with 74 genes in the GO term angiogenesis pathway. **Table 1.** A list of genes exhibiting upregulation and downregulation in AZGP1-OV PC3 tumors in contrast to vector control PC3 tumors.

**Additional file 2:** Proteomics data of PC3 and DU145 vector control and AZGP1-OV tumors.

## Data Availability

All data and materials are included in the paper, and raw data in this study will be available upon request to the corresponding author. Proteomic data are available as additional file 2.

## Abbreviations

AZGP1: Zinc-alpha-2-glycoprotein
HUVECs: Human umbilical vein endothelial cells
GO: Gene Ontology
MHC: Major histocompatibility complex
PCa: Prostate cancer
PCR: Polymerase chain reaction
PSA: Prostate specific antigen
RT-PCR: Reverse-transcription polymerase chain reaction
RT-qPCR: Real-time quantitative polymerase chain reaction
TMA: Tissue microarray
