# Supplementary material for: AZGP1 deficiency promotes angiogenesis in prostate cancer
Source: J Transl Med. 2024 Apr 24;22:383. doi: 10.1186/s12967-024-05183-x (PMC11044612; doi:10.1186/s12967-024-05183-x)
Supplement: Supplementary file 1 — Additional file 1: Figure S1. (A) Overexpression of AZGP1 in AZGP1-OV 22Rv1 cells did not affect cell proliferation. (B) Western blot confirming increased AZGP1 protein expression in 22Rv1 AZGP1-OV cells. (C) Overexpressing of AZGP1 does not affect colony formation in 22Rv1 cells. (D) Quantification of colony formation in control and AZGP1-OV 22Rv1. (E) Rpresentative images of 22Rv1 vector control and 22Rv1 AZGP1-OV tumor xenografts. (F) Tumor weight of 22Rv1 vector control and 22Rv1 AZGP1-OV. (G) Representative images showing similar tumor morphology by H & E, validation of AZGP1 overexpression, and CD31 staining microvessels in 22Rv1-AZGP1-OV tumors and control tumors. Figure S2. Relative expression levels of (A) ANXA1, (B) HSPB1, (C) GPX1, (D) RNH1, (E) COL18A1, (F) YWHAZ, (G) SERPINE1 in PC3 control and AZGP1-OV tumors, determined by mass spectrometry. Figure S3. Relative expression levels of (A) GPX1, (B) RNH1, (C) COL18A1, (D) YWHAZ, (E) PDCD6 in DU145 control and AZGP1-OV tumors, determined by mass spectrometry. Figure S4. Genes correlated with AZGP1 expression levels in TCGA-PRAD overlapped with 74 genes in the GO term angiogenesis pathway. Table 1. A list of genes exhibiting upregulation and downregulation in AZGP1-OV PC3 tumors in contrast to vector control PC3 tumors. [file 12967_2024_5183_MOESM1_ESM.docx]

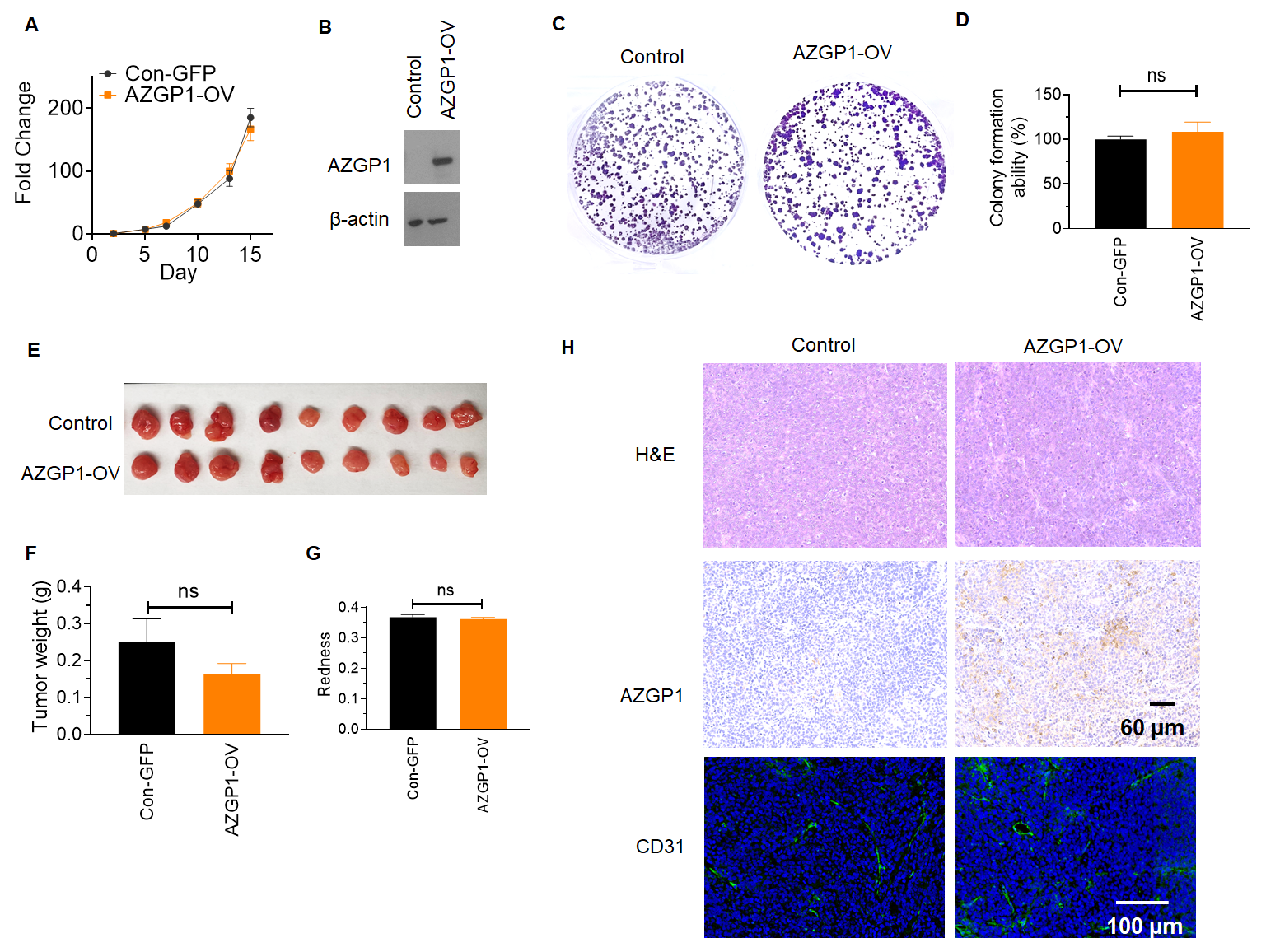


Figure S1. (A) Overexpression of AZGP1 in AZGP1-OV 22Rv1 cells did not affect cell proliferation. (B) Western blot confirming increased AZGP1 protein expression in 22Rv1 AZGP1-OV cells. (C) Overexpressing of AZGP1 does not affect colony formation in 22Rv1 cells. (D) Quantification of colony formation in control and AZGP1-OV 22Rv1. (E) Rpresentative images of 22Rv1 vector control and 22Rv1 AZGP1-OV tumor xenografts. (F) Tumor weight of 22Rv1 vector control and 22Rv1 AZGP1-OV. (G) Representative images showing similar tumor morphology by H & E, validation of AZGP1 overexpression, and CD31 staining microvessels in 22Rv1-AZGP1-OV tumors and control tumors.


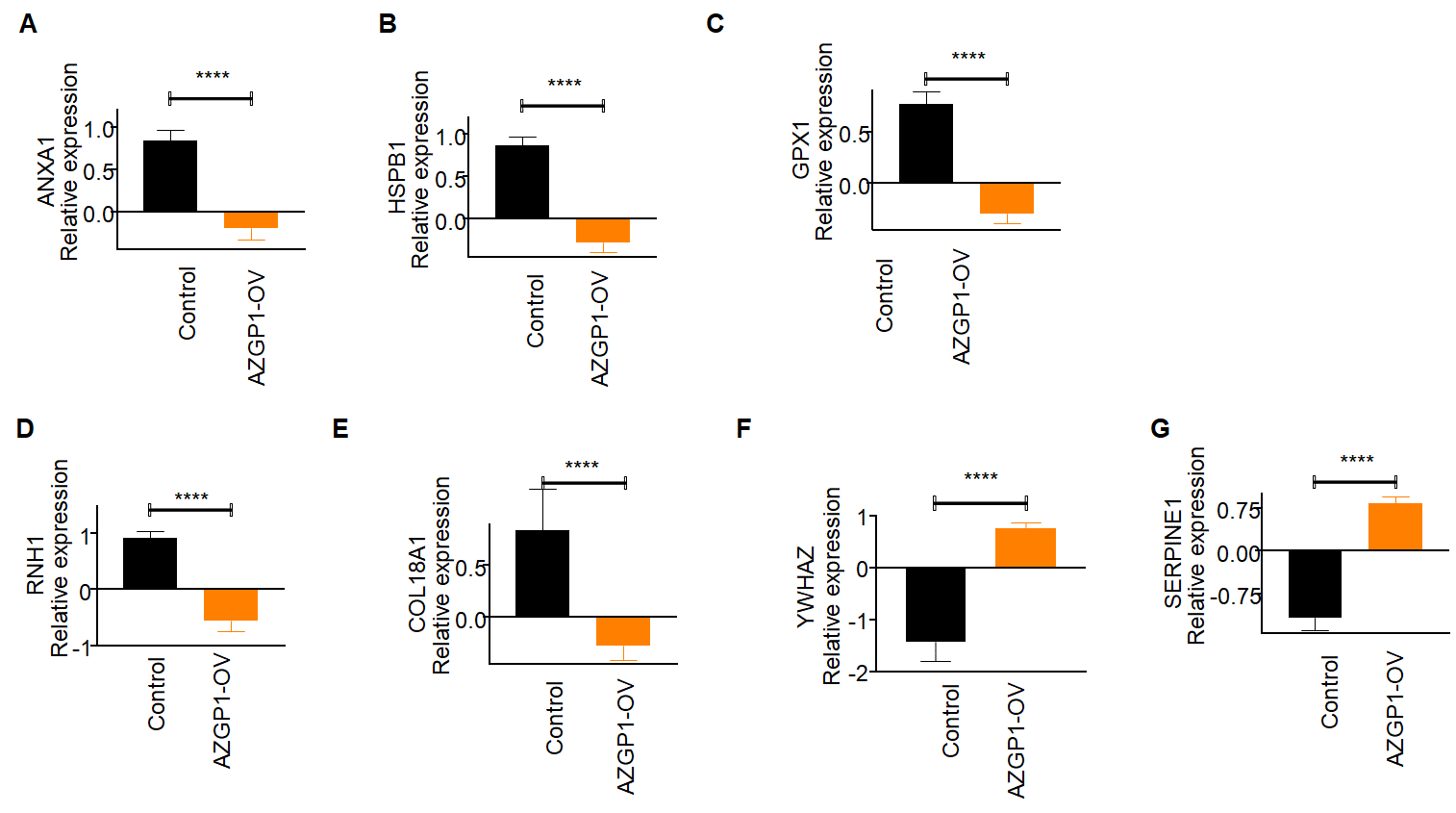


Figure S2. Relative expression levels of (A) ANXA1, (B) HSPB1, (C) GPX1, (D) RNH1, (E) COL18A1, (F) YWHAZ, (G) SERPINE1 in PC3 control and AZGP1-OV tumors, determined by mass spectrometry.


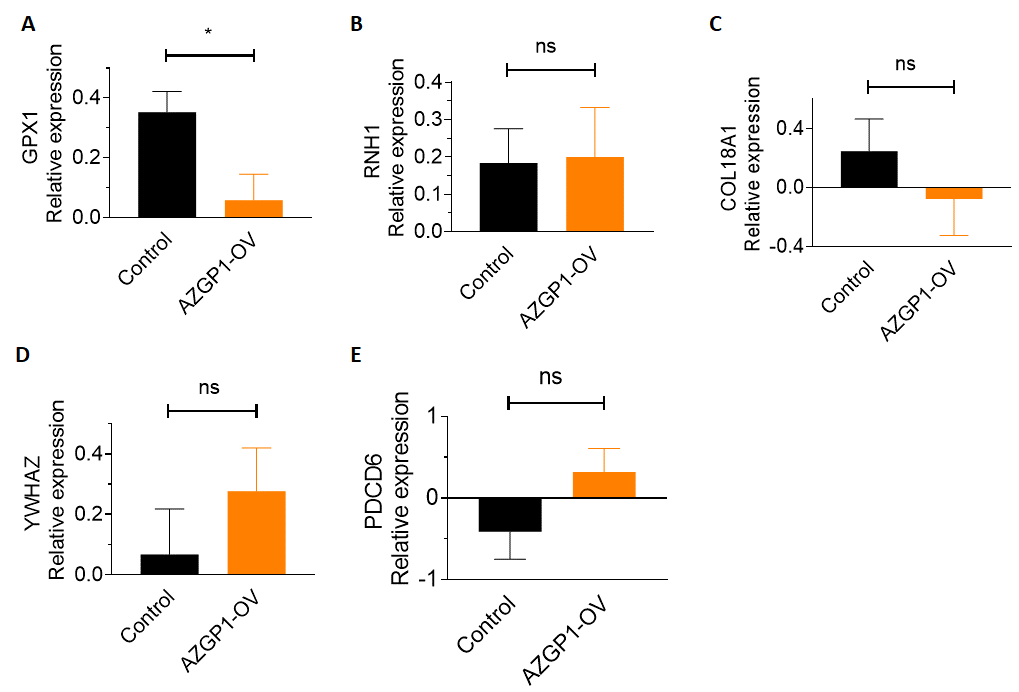


Figure S3. Relative expression levels of (A) GPX1, (B) RNH1, (C) COL18A1, (D) YWHAZ, (E) PDCD6 in DU145 control and AZGP1-OV tumors, determined by mass spectrometry.

Figure S4 Genes correlated with AZGP1 expression levels in TCGA-PRAD overlapped with 74 genes in the GO term angiogenesis pathway.

Table 1 A list of genes exhibiting upregulation and downregulation in AZGP1-OV PC3 tumors in contrast to vector control PC3 tumors

| Gene Name | log2 (fc) | -log(p) |
| --- | --- | --- |
| SERPINE1 | 2.003062 | 6.067896 |
| YWHAZ | 2.180557 | 4.413624 |
| EPHA2 | 1.497037 | 4.319662 |
| PDCD6 | 1.519989 | 4.245097 |
| GTF2I | 1.112674 | 3.65412 |
| PKM | 1.54521 | 3.540235 |
| HK2 | 1.340204 | 3.489449 |
| PXN | 0.9847 | 3.406086 |
| PGK1 | 1.048806 | 3.379259 |
| CTNNB1 | 2.073469 | 3.304442 |
| PNPLA6 | 0.942191 | 3.058408 |
| ROCK2 | 1.660978 | 2.996045 |
| HMGB1 | 1.667595 | 2.879129 |
| ANXA2 | 0.96719 | 2.8454 |
| PARVA | 1.207348 | 2.719219 |
| CLIC4 | 1.66429 | 2.523227 |
| CDC42 | 1.9547 | 2.473512 |
| MYH9 | 0.663242 | 2.229714 |
| MAPK14 | 1.09679 | 2.214652 |
| RHOA | 1.024672 | 2.034992 |
| STAT1 | 0.426674 | 1.895267 |
| ACTG1 | 4.528359 | 1.678165 |
| FN1 | 0.82456 | 1.653815 |
| EPN1 | 1.16068 | 1.649192 |
| PLCG1 | 0.901481 | 1.619949 |
| FLNA | 0.612487 | 1.532775 |
| NRP1 | 0.789286 | 1.494189 |
| RNF213 | 1.560479 | 1.455726 |
| TJP1 | 0.459119 | 1.428735 |
| MMP9 | -7.1448 | 10.49804 |
| GPX1 | -1.06614 | 5.711038 |
| HSPB1 | -1.13777 | 5.669291 |
| COL18A1 | -1.11887 | 5.308254 |
| RNH1 | -1.48418 | 5.130718 |
| CALD1 | -1.98011 | 4.40121 |
| ANXA1 | -1.03436 | 4.367334 |
| ANPEP | -1.38031 | 4.095995 |
| ANXA3 | -0.8228 | 3.756164 |
| PLAU | -2.02011 | 3.697024 |
| ERAP1 | -1.76457 | 3.536119 |
| COL4A1 | -1.29719 | 3.122154 |
| LOXL2 | -2.50268 | 2.953424 |
| THBS1 | -1.03145 | 2.911205 |
| IL18 | -2.93753 | 2.890796 |
| TNFAIP2 | -2.83203 | 2.877164 |
| NAXE | -1.05232 | 2.828925 |
| PRCP | -1.83404 | 2.379302 |
| LGALS3 | -0.71302 | 2.275703 |
| DCN | -2.53527 | 2.259456 |
| MYDGF | -0.73867 | 2.187877 |
| CIB1 | -1.25718 | 1.974782 |
| EGLN1 | -1.50547 | 1.966979 |
| COL4A2 | -0.95185 | 1.906903 |
| STAT3 | -1.15305 | 1.606961 |
